# Revisiting Tversky's diagnosticity principle

**DOI:** 10.3389/fpsyg.2014.00875

**Published:** 2014-08-12

**Authors:** Ellen R. K. Evers, Daniël Lakens

**Affiliations:** ^1^Department of Social Psychology, TIBER, Tilburg UniversityTilburg, Netherlands; ^2^Department of Human-Technology Interaction, Eindhoven University of TechnologyEindhoven, Netherlands

**Keywords:** replication, similarity, diagnosticity, judgments, categorization

## Abstract

Similarity is a fundamental concept in cognition. In 1977, Amos Tversky published a highly influential feature-based model of how people judge the similarity between objects. The model highlights the context-dependence of similarity judgments, and challenged geometric models of similarity. One of the context-dependent effects Tversky describes is the diagnosticity principle. The diagnosticity principle determines which features are used to cluster multiple objects into subgroups. Perceived similarity between items within clusters is expected to increase, while similarity between items in different clusters decreases. Here, we present two pre-registered replications of the studies on the diagnosticity effect reported in Tversky (1977). Additionally, one alternative mechanism that has been proposed to play a role in the original studies, an increase in the choice for distractor items (a substitution effect, see Medin et al., 1995), is examined. Our results replicate those found by Tversky (1977), revealing an average diagnosticity-effect of 4.75%. However, when we eliminate the possibility of substitution effects confounding the results, a meta-analysis of the data provides no indication of any remaining effect of diagnosticity.

## Introduction

In 1977, Amos Tversky published a highly influential paper on features of similarity. He critiqued geometric models of similarity, and proposed an alternative approach known as the contrast model. Tversky's contrast model is built on feature matching processes where similarity judgments are based on the contrast between common and distinctive features of two stimuli, and where the salience of these features is, among other things, dependent on the context. The diagnosticity principle, the focus of the current replication project, is one of the mechanisms through which similarity judgments become context-dependent.

Tversky (1977) argues that the salience of features is determined by intensive and diagnostic factors. Intensive factors are those that increase the intensity of the feature, such as size, brightness, and clarity. Diagnostic factors are context-dependent and refer to the importance based on those features. Tversky illustrates the diagnosticity of features with the following example: “the feature ‘real’ has no diagnostic value in a set of actual animals since it is shared by all actual animals and hence cannot be used to classify them. This feature, however, acquires considerable diagnostic value if the object set is extended to include legendary animals, such as a centaur, a mermaid, or a phoenix” (Tversky, 1977, p. 342).

When people sort sets of objects into sub-sets or clusters, these clusters are usually created such that they maximize similarity of objects within the cluster and dissimilarity of objects between the clusters. Because the addition of other items to a set of objects can alter how people sort objects into clusters, the diagnostic value of features on which these clusters are based will change depending on the overall context. This will in turn influence the judged similarity between objects that share these diagnostic features. According to Tversky's diagnosticity principle, features that are used to cluster stimuli into subgroups have a higher diagnostic value and will therefore affect similarity judgments more than features that are not used to create clusters. Features shared within a cluster will increase the similarity between objects in that cluster, whereas features that differ across clusters will decrease the similarity between objects from different clusters.

The diagnosticity principle is especially relevant when people are asked how similar two stimuli are, and the question does not specify any specific feature space to base the similarity judgment on. Tversky's model of similarity is a major contribution to the field of psychology, and even though follow-up research has expanded upon the model (see for example Gentner and Markman, 1997), the main premises of the model are still accepted today, as indicated by the large number of citations (6242 in Google Scholar as of June, 2014). In other words, Tversky's paper led to a fundamental change in how scientists think about similarity judgments (for a discussion, see Goldstone and Son, 2005). The diagnosticity principle is especially interesting for psychologists, because it revealed how geometric models of similarity failed to take into account that human cognition is inherently context-dependent. It is the context that determines how different features of stimuli are weighed in similarity judgments.

Tversky's diagnosticity principle contributed to what is currently understood to be a basic principle of similarity judgments. It is therefore remarkable that only a modest number of studies have tried to (conceptually) replicate the diagnosticity effect. Furthermore, whereas the original studies have yielded clear results, follow up studies do not allow unequivocal conclusions. All the replication attempts either test the diagnosticity effect in a different way (which makes it difficult to compare effect sizes between studies), or suffer from methodological or data-analytical problems. Therefore, there is a general imbalance between the theoretical importance of the diagnosticity effect, and the amount of empirical support for its existence.

## Studies on diagnosticity: tversky (1977)

**Figure 1:**
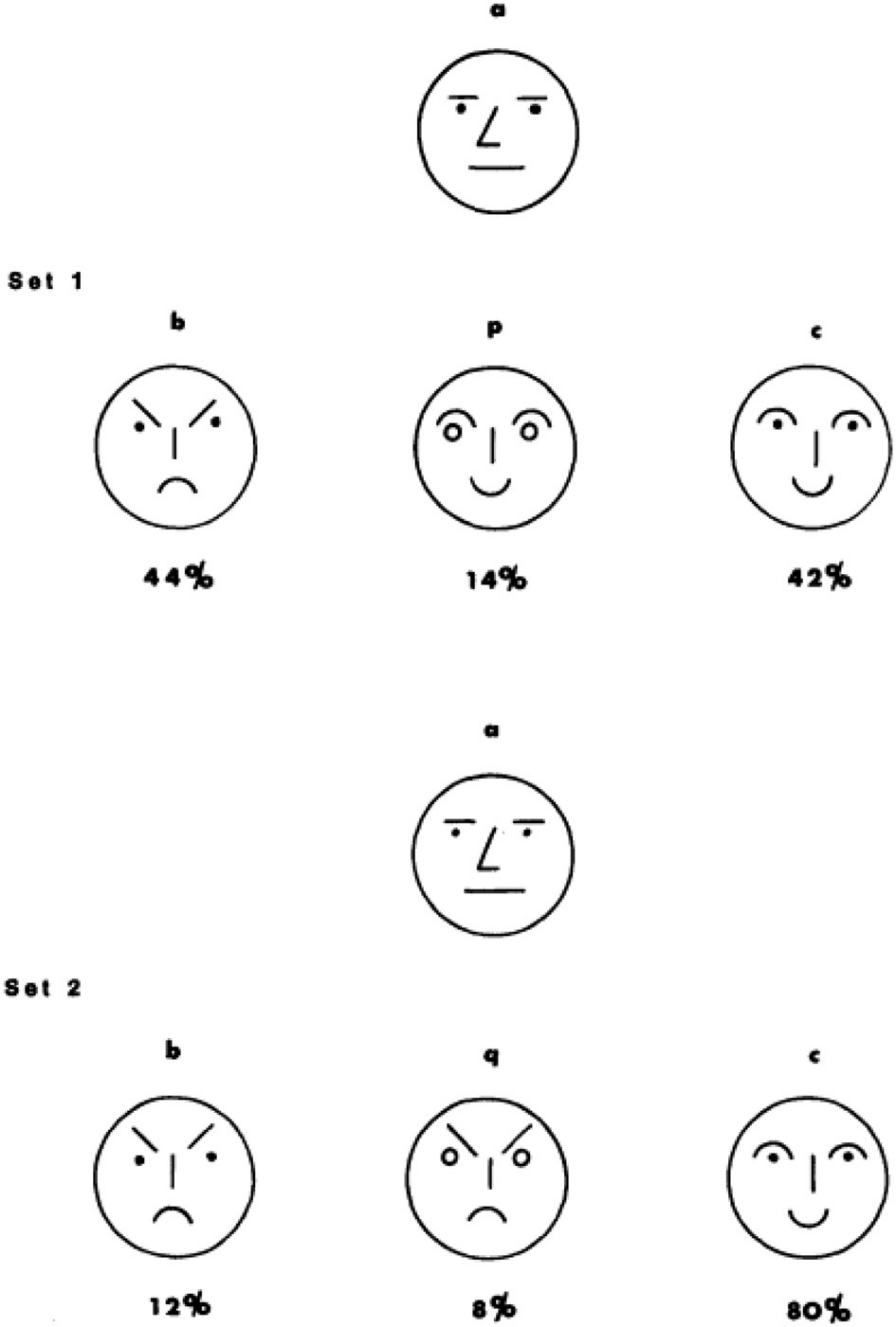
**Stimuli used in Experiment 1 (Tversky, 1977)**.

Tversky reports two different studies that provide support for the diagnosticity effect. The first study reported in Tversky (1977) uses a straightforward paradigm. First, participants were shown a group of four faces (see Figure 1, below).

This group of faces always consisted of a neutral (*a*, the target stimulus on the top), frowning (*b*), and smiling (*c*) face. For half of the participants (condition 1) the fourth face was smiling (*p*), for the other half (condition 2) it was frowning (*q*). Participants were asked to split the four faces into two groups of two faces. As expected, when the fourth face was smiling most participants grouped *c*&*p* (smiling) and *a*&*b* (non-smiling), but when the fourth face was frowning participants grouped *b*&*q* (frowning) and *a*&*c* (non-frowning).

According to the diagnosticity principle, smiling vs. non-smiling had a greater diagnostic value in the first set of faces, but frowning vs. non-frowning was a more diagnostic classification feature in the second set of faces. Subsequently, a different group of participants (*N* = 50) was asked to pick the face that most resembled the target face (*a*) from a group of *b, p*[*q*], and *c*. Mirroring the choices participants made when classifying the four faces into two subgroups, participants were more likely to pick the frowning face as most similar to the neutral target face when the other two faces were smiling (condition 1), as compared to when two of the three faces were frowning (condition 2, for the choice proportions, see Figure 1). The reverse was true for the smiling face, which was more likely to be picked as similar to the neutral face *a* in condition 2 as compared to condition 1. The diagnosticity principle was thus represented by the proportion selecting the frowning face in condition 1 minus the proportion selecting the frowning face in condition 2, as well as the proportion selecting the smiling face in condition 2 minus the proportion selecting the frowning face in condition 1.

**Figure 2:**
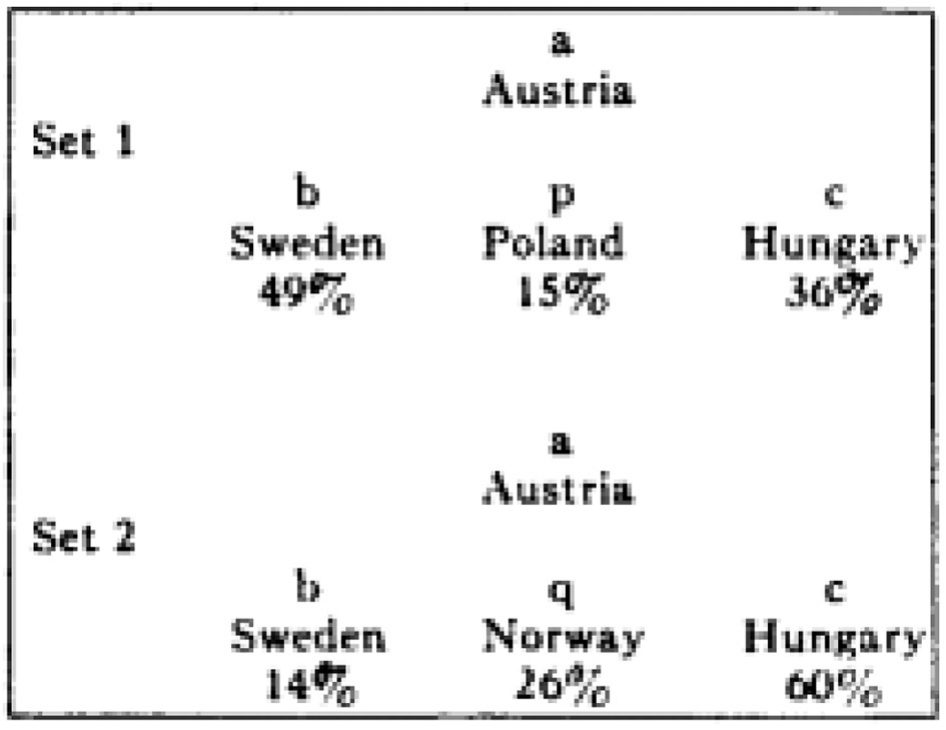
**Example of stimulus used in Experiment 4 (Tversky and Gati, 1978)**.

Study 2 on the diagnosticity principle, (described in more detail as Experiment 4 in Tversky and Gati, 1978) is a conceptual replication using semantic stimuli. Instead of using sets of four faces, sets of four countries were used. First, an independent group of participants was asked to classify the 4 countries into two groups (although as opposed to Experiment 1, no longer necessarily into two groups of two). Three countries were the same in each condition (*a, b*, & *c*) but the fourth varied depending on the condition (*p*/*q*). Instead of a single trial experiment (as in Experiment 1), 20 sets of countries were created. After this categorization task, a new group of participants (*N* = 33) was asked to make similarity judgments (as in Experiment 1, see Figure 2 for an example trial).

It was expected that similarity judgments would follow the same pattern as the categorizations made by the previous group of participants, and this pattern of results was indeed observed. Whereas in Experiment 1 a single trial consisting of novel and visual stimuli is used (i.e., schematic faces), in Experiment 2 diagnosticity is based on existing, semantic knowledge (i.e., characteristics of countries), and multiple trials were used. Other than that, the experiments closely resemble each other.

## Substitution effect

A problem with these studies, as explained by Medin et al. (1995), is that a change in choice proportion does not necessarily reflect a diagnosticity effect but could also be the result of a substitution effect. The substitution effect is most often discussed in areas of consumer choice when two goods have positive cross-elasticity such as chocolate ice-cream and vanilla ice-cream. The introduction of one item into the market (i.e., chocolate ice-cream) reduces demand for the other item (vanilla ice-cream). Medin and colleagues propose something similar may have occurred in the studies by Tversky (1977) if it was the case that the distractor stimulus (the item varying across condition, *p* or *q*) drew more choices away from one option than from the other.

**Figure 3:**
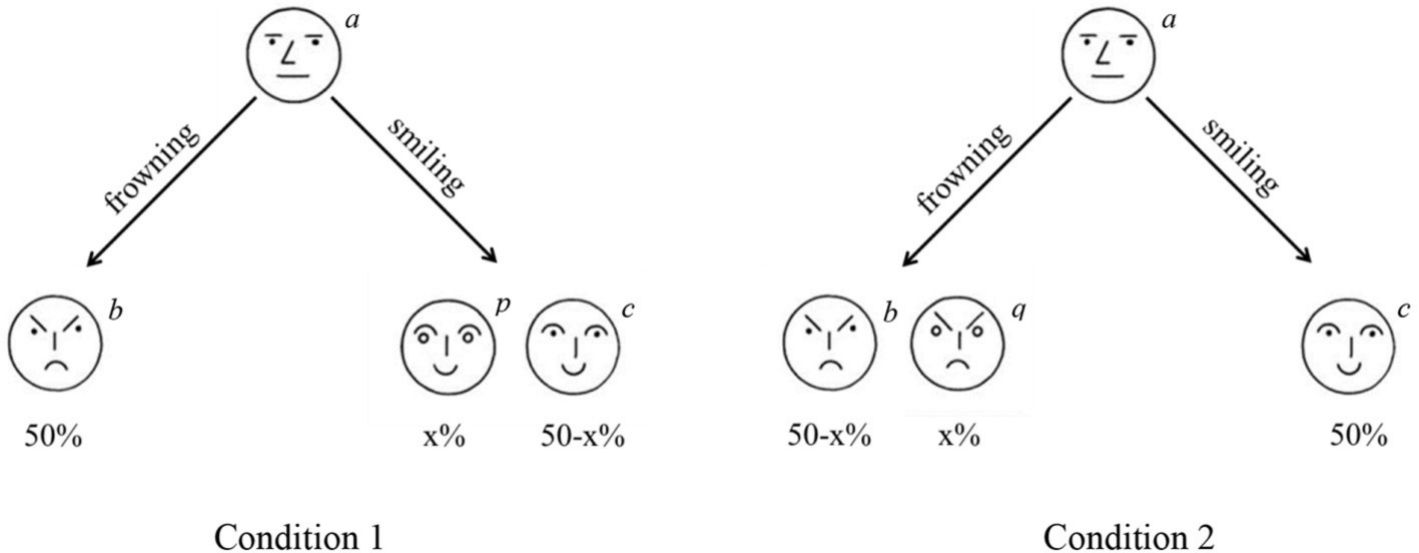
**Illustration of substitution effect**. In the situation below more people would choose the face on the left (*b*) in condition 1 as compared to condition 2 because choices for a frowning face are spread over two options in condition 2.

This may be easiest to illustrate with a (hypothetical) example shown in Figure 3. Imagine that in the population 50% of the people believe that a neutral face (see Figure 3) is most similar to a frowning face. The other 50% believe it is most similar to smiling face. In condition 1, this means that 50% of the participants judge face *b* (frowning) is most similar to face *a* (neutral), since face *b* is the only frowning face in the choice-set. The other 50% of the participants could spread their choice over face *p* and face *c* since both those faces are smiling. In condition 2, 50% of the participants would judge face *c* as most similar to face *a* since it is the only smiling face. The remaining 50% of the participants could spread their choices over both face *b* and face *q*, both of which are frowning. As a result, choices differ between condition 1 and 2, but this difference is not necessarily because the inclusion of distractor face *p*/*q* changes similarity judgments of face *b* or *c*, but merely because one group of people (e.g., those who believe frowning is most like smiling) spread their choices over two options. Differences in choice due to substitution-effects would occur in the same direction as differences predicted on the basis of a diagnosticity effect. As a result, it is unclear whether the data provided by Tversky (1977) is truly evidence for a diagnosticity effect or merely represents substitution-effects.

Substitution effects can be eliminated by ranking the choice-options rather than selecting from them (Medin et al., 1995). To reiterate, the diagnosticity-principle would predict that in Figure 3 the inclusion of a smiling face (*p*) would increase the perceived similarity between the frowning face (*b*) and the neutral face (*a*). Inclusion of a frowning face (*q*), would increase perceived similarity between the smiling face (*c*) and the neutral face (*a*). A substitution effect predicts that inclusion of a smiling face (*p*) draws choices away from the other smiling face (*c*), but crucially, does not predict that people would perceive the frowning face as (b) more similar to the neutral face (*a*). As such, both a substitution-effect as well as a diagnosticity-effect would result in a relative increase in choices for *b* in condition 1 as compared to condition 2. However, only a diagnosticity-effect would result in *b* being ranked above *c* more in condition 1 as compared to condition 2.

## Close and conceptual replications

Despite the straightforward experimental method and the theoretical influence the original experiments have had, there are remarkably few replication attempts. Even more striking is that all attempts we were able to find in the literature could either not be directly compared with the original studies by Tversky or had no strong evidential value for a diagnosticity effect.

## Direct replications

A literature search revealed one published close replication, in which participants from two different cultures (China and Australia) performed the original experiment (Zhou et al., 2005). The results of this experiment are difficult to interpret, since the number of participants selecting each item is not reported. Furthermore, because the authors are interested in whether their participants differ from Israeli participants, the crucial test for the diagnosticity-effect (comparing choices in set 2 with choices in set 1) is not reported. Instead, the authors test whether the choice proportions by the Chinese and Australian participants were different from each other, and different from Tversky's original results. The original authors have kindly provided the choice-data which allow us to calculate whether a diagnosticity effect was found in their study. For the Chinese participants, 6/30 selected face *b* as most similar to the target in condition 1 and 7/30 participants selected face b in condition 2. This difference is not significant, χ^2^_(1, *N* = 60)_ = 0.10, *p* = 0.75. In condition 2, 21/30 participants selected face *c* to be most similar whereas in condition 1 this was only 14/30 participants. This difference was marginally significant, χ^2^_(1, *N* = 60)_ = 3.36, *p* = 0.07. For the Australians, 14/31 selected face *b* to be most similar in condition 1 and 13/34 in condition 2. This was not significant, χ^2^_(1, *N* = 65)_ = 0.32, *p* = 0.57. Furthermore, in condition 2, 21/34 selected face c to be most similar while only 14/31 did so in condition 1. This difference was also not significant, χ^2^_(1, *N* = 65)_ = 1.80, *p* = 0.18. Thus, whereas these data do indeed show that choices differ across cultures, which was the claim of the paper, they do not support the presence of a diagnosticity effect.

The only other direct replications of Study 1 we know of are two unpublished datasets collected by the second author. This data was collected in an online adaptation of the original experiment (as in Study 1b and Study 2b) as part of an assignment in an introduction to psychology class (similar to the original studies by Tversky) in 2012 and 2013. The sample sizes were relatively small (2012: *n*_1_ = 45, *n*_2_ = 41, 2013: *n*_1_ = 63, *n*_2_ = 70, but the results (available from http://osf.io/e6cr3/) were still insightful since the null-effect was a trend in opposite direction of the hypothesized (and previously found) effect. Given the lack of an effect in the replication study, we should seriously consider the possibility that Tversky's (1977) effect-size is an overestimation.

## Conceptual replications

**Figure 4:**
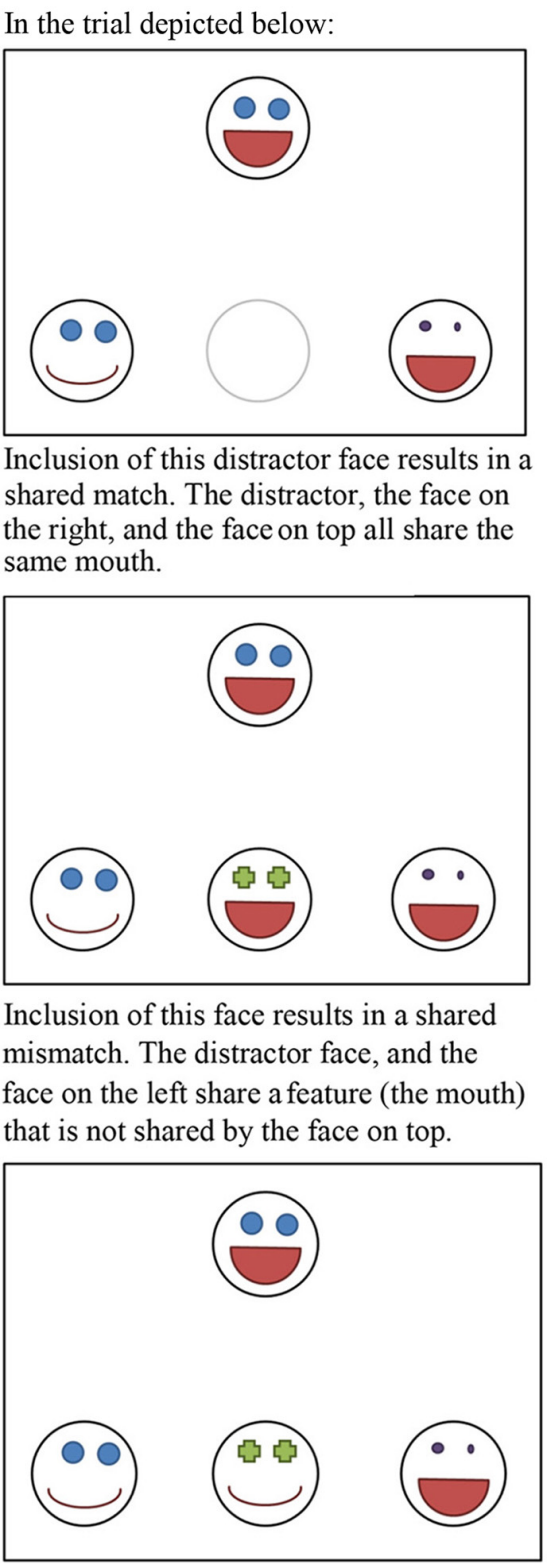
**Illustration of a shared-match and a shared-mismatch**.

A conceptual replication (Goldstone et al., 1997, Experiment 2A and 2B) used schematic faces similar to those used by Tversky (1977) with one extension. On some trials, the distracter and one of the other two choice options shared a feature that did not match the comparison stimulus (a shared mismatch), while on other trials the distracter shared a feature that was also possessed by the comparison stimulus (a shared match, see Figure 4). Because shared mismatches are likely to be categorized separately from the target, these would be expected to decrease similarity between the shared-mismatching items and the target item. Shared matches are likely to be categorized together with the target, and would therefore increase similarity. Goldstone et al. (1997) report a difference in choice-proportions such that for shared matches participants are slightly more likely to choose the option that shares a feature with item *a* (the target item portrayed on top) than the option not sharing a feature with *a* (i.e., in Study 2a 50.8% chooses the option sharing a feature while 48.5% chooses the option not sharing a feature, while in Study 2b 51% chooses the option sharing a feature while 48.5% chooses the option not sharing a feature in study 2b). This effect is opposite from what would be expected on the basis of a categorization-based diagnosticity account, but are in line with a variability-based diagnosticity account proposed by the authors.

The second conceptual replication, conducted by Medin and Kroll (as discussed in Medin et al., 1995), used geometrical shapes, and attempted to examine whether the diagnosticity effect occurred above and beyond a substitution effect. Rather than examining the change in choice-proportion, as done by Tversky (1977), they count the number of trials in which the diagnostic option (the option that shares a relationship with the target that is not shared by the other options) is ranked as being more similar to the target than one of the non-diagnostic options (using the relative ordering task also used in the current studies), and report the diagnostic option was selected in 55% of the trials (chance would predict this to happen in 50% of the trials). This result suggests that there is indeed a diagnosticity-effect beyond substitution.

## Overview of the current studies

As the preceding section illustrates, support for the diagnosticity effect is mixed and limited. We know of six close replication attempts. Of these six datasets, four are interpretable but fail to find an effect (two datasets by the second author available from http://osf.io/e6cr3/ and Zhou et al., 2005). The final two experiments (Medin and Kroll in Medin et al., 1995; Goldstone et al., 1997) do replicate a diagnosticity effect, but this effect is much smaller than the original diagnosticity effect found by Tversky (1977). These differences in effect size could be a consequence of the differences in methodology, but it is also possible that Tversky's original findings are by large the result of a substitution effect misinterpreted as a diagnosticity effect. Because of the importance of the diagnosticity effect, and the limited empirical evidence for it, we believe that the original work by Tversky (1977) is an excellent candidate for replication. Therefore, we set out to perform a pre-registered replication of both Study 1 and Study 2 reported in Tversky (1977).

To examine the possibility of a substitution effect we extended the original studies by adding questions after the original procedure used by Tversky and Gati (1978), designed to distinguish between the substitution effect and the diagnosticity effect. We first asked participants to choose the most similar target in a close replication of Tversky (1977), and subsequently used a ranking task to distinguish between a diagnosticity effect and a substitution effect (cf. Medin et al., 1995). This means that it is possible to successfully replicate the data pattern observed by Tversky (1977), while concluding there is no evidence for a diagnosticity effect, if the observed effect disappears after eliminating a substitution effect.

We used larger sample sizes for more accurate estimates of effect-sizes as well as to guarantee sufficient statistical power even when the effect size reported is an overestimation of the true effect size. Because the small number of replications does not provide a reasonably accurate meta-analytical point-estimate for the true effect size, we used at least 2.5 times the original *N* following recommendations in Simonsohn (2013), to have approximately 80% power to detect an effect of the size the original studies had 33% power to detect. This results in a minimum of 125 participants in the categorization task for Study 1a and 1b, 250 participants in the similarity task for Study 1a and 1b, 125 participants in the categorization task for Study 2a and 2b, and 175 participants in the similarity task in Study 2a and 2b. Note that the categorization-task itself is not a replication-study, but that categorization is a necessary condition for the diagnosticity-effect to emerge. All materials, data, and the replication-proposal can be found on the OSF-framework: http://osf.io/e6cr3/. All analyses and exclusions presented below were planned unless explicitly noted.

## Study 1

Study 1 was a close replication of the first study on the diagnosticity principle in Tversky (1977). In total two replications of Study 1 were performed. In Study 1a Dutch students from Eindhoven University of Technology and Tilburg University performed a paper and pencil version of the task. In Study 1b a computerized version of the task was performed by an American population of Amazon mTurk workers. For both replication studies one group of participants performed the categorization task and another group of unique participants performed the similarity judgments.

### Study 1a

#### Method

***Categorization task***. Two-hundred students from Tilburg University were approached on campus to complete the categorization task. They were randomly assigned to one of two conditions in which they saw the four original faces used by Tversky(1977, see Figure 1) displayed on a horizontal line, and were asked to assign the four faces to two different groups. Both versions consisted of one neutral, one frowning and one smiling face. In the first condition the fourth face was another (slightly different) smiling face (*p*). In the second condition, the fourth face was another (slightly different) frowning face (*q*). Placement of the neutral face was counterbalanced between participants.

***Similarity task***. Two-hundred-fifty-four students from Eindhoven University of Technology were approached on campus and randomly assigned to one of two conditions in which they were presented with four drawn faces (see Figure 1). In both conditions, the face on top always had a neutral expression (*a*), the face on the left was always frowning (*b*) and the face on the right was always smiling (*c*). The face in the middle of the bottom row varied per condition. In condition 1 it was another smiling face (*p*) while in condition 2 it was another frowning face (*q*). On the first page, participants were asked to indicate which of the faces on the bottom row was most similar to the face on top (*a*), similar to Tversky (1977). Extending the procedure used by Tversky (1977), on the second page participants saw the same four faces and were asked to rank the faces on the bottom row from most similar (#1) to least similar (#3) to the face on top. Following Medin et al. (1995), this second question allows us to distinguish between a diagnosticity effect and a substitution effect.

#### Results

***Categorization task***. Similar to Tversky (1977), a large proportion of the students (81/99, 81.8%) in condition 1 grouped the smiling faces (*p* and *c*) together, and grouped the neutral face (*a*) together with the frowning face (*b*). In condition 2, a large proportion of the students (78/100, 78%) grouped the frowning faces (*b* and *p*), and the neutral and smiling face (*a* and *c*) together.

***Similarity task***. Based on the results of the categorization task, participants in condition 1 were expected to be more likely to indicate the neutral face (*a*) being similar to the frowning face (*b*) as compared to participants in condition 2, but this was not the case (38/12, 29.9% in condition 1, 46/127, 36.2% in condition 2, the diagnosticity effect was calculated by subtracting the choice-proportion in condition 2 from the choice proportion in condition 1 resulting in a diagnosticity effect of −6.30%, χ^2^_(1, *N* = 254)_ = 1.14, *p* = 0.29, Cramer's *V* = 0.067. Similarly, participants in condition 2 were expected to be more likely to indicate the frowning face (*c*) to be more similar to the neutral face (*a*) as compared to participants in condition 1, but this was also not the case (80/127 in condition 1, 81/127 in condition 2, diagnosticity effect 0.79%, χ^2^_(1, *N* = 254)_ = 0.02, *p* = 0.90, Cramer's *V* = 0.008.

Even though no significant effect was found, analyzing the rank-orders instead of choice is a more accurate estimate of the diagnosticity-effect. Therefore, and for consistency with the following three studies, we also calculated the diagnosticity effect eliminating possible substitution-effects. To do this, we followed the procedure by Medin et al. (1995), and recoded the rankings into a dichotomous variable indicating whether the frowning face (*b*) was judged to be more similar to the neutral face (*a*) than the smiling face (*c*), or indicating that the smiling face (*c*) was more similar to the neutral face (*a*) than the frowning face (*b*)^1^. 42/126 Participants^2^ in condition 1, and 46/127 participants in condition 2 judged the sad face (*b*) as more similar to the neutral face (*a*) than the smiling face (*c*). Analyzing the data using rank-orders also resulted in a non-significant diagnosticity effect of 2.89%, χ^2^_(1, *N* = 253)_ = 0.23, *p* = 0.63, Cramer's *V* = 0.03. See Table 1 for an overview of these results.

**Table 1:** **Results Study 1a and 1b**.

**Sample**	**Categorization**		**Similarity**		**Eliminating substitution**
	***b*(*p*) – *b*(*q*) (%)**	***c*(*q*) – *c*(*p*) (%)**	***b*(*p*) – *b*(*q*) (%)**	***c*(*q*) – *c*(*p*) (%)**	***b* > *c*(*p*) – *b* > *c*(*q*) (%)**
Dutch students	74.82	65.88	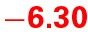	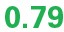	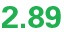
mTurk	86.49	91.89	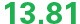	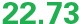	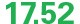

*The categorization and similarity results represent the difference (in percentages) in choices for targets b and c between the two conditions (p and q). The right most column represents the average difference (in percentages) between the two conditions of the percentage of times option b was ranked higher than c. Results in green are in the direction predicted by a diagnosticity effect, those in red are opposite to the expected direction*.

### Study 1b

#### Method

***Categorization task***. One-hundred-and-fifty-five workers on mTurk participated in this task in return for $0.10. They were randomly assigned to one of two conditions in which they saw four drawings of a face and were asked to assign them to two different groups, as in Study 1a. Placement of the neutral face was counterbalanced between participants.

***Similarity task***. Two-hundred-and-two workers on Amazon mTurk participated in this task in return for $0.16 and were randomly assigned to one of two conditions^3^. As in Study 1a they were presented with four drawn faces (see Figure 1), and were first asked to choose which face was most similar to the neutral face depicted on top. Subsequently, participants ranked the faces in order of similarity as in Study 1a.

#### Results

***Categorization task***. Similar to Tversky (1977) and Study 1a, a large proportion of the participants (66/75, 88%) in condition 1 grouped the smiling faces (*p* and *c*) together, and grouped the neutral face (*a*) together with the frowning face (*b*). In condition 2, a large proportion of the participants (64/72, 88.9%) grouped the frowning faces (*b* and *p*), and the neutral and smiling face (*a* and *c*) together.

***Similarity task***. The similarity task consisted of two parts. First, participants indicated which face in the bottom row was most similar to the neutral face (*a*) presented on top. Subsequently, participants ranked the faces in the bottom row in order of most similar to least similar to the face on top. Participants in condition 1 were expected to be more likely to indicate the neutral face (*a*) being similar to the frowning face (*b*) as compared to participants in condition 2, and this was indeed the case (41/93 in condition 1, 33/109 in condition 2, diagnosticity effect = 13.81%, χ^2^_(1, *N* = 202)_= 4.12, *p* = 0.04, Cramer's *V* = 0.143. Similarly, participants in condition 2 were expected to be more likely to indicate the smiling face (*c*) to be more similar to the neutral face (*a*) as compared to participants in condition 1, which also was confirmed (74/109 in condition 2, 42/93 in condition 1, diagnosticity effect 22.73%, χ^2^_(1, *N* = 202)_ = 10.6, *p* = 0.001, Cramer's *V* = 0.229.

To investigate how much of this shift can be explained by a substitution effect, we also analyzed the rankings. Similar to Study 1a, rankings were recoded into either indicating that the frowning face (*b*) was more similar to the neutral face (*a*) than the smiling face (*c*), or indicating that the smiling face (*c*) was more similar to the neutral face (*a*) than the frowning face (*b*). In the first condition, 47/93 participants judged the sad face (*b*) as more similar to the neutral face (*a*) than the smiling face (*c*). In the second condition, only 35/106 participants did so^4^. This reduced, but did not eliminate the diagnosticity effect; 17.52%, χ^2^_(1, *N* = 199)_ = 6.28, *p* = 0.01, Cramer's *V* = 0.178. In other words, if we directly replicate the analysis by Tversky (1977) on our data, we find evidence consistent with his original findings. Eliminating a confounding factor present in the original study reduces the effect but still provides evidence for a diagnosticity effect. See Table 1 for an overview of all results.

Where Study 1a does not seem to provide any support for the existence of a diagnosticity effect, Study 1b provides support for the diagnosticity effect, even when eliminating substitution effects. The effect size is in the replication study is smaller than the effect size in the original study reported by Tversky (1977), who found a difference of 32% in choices for *b* and 38% in choices for *c*, whereas we only found a 17.5% change in Study 1b, and a negative diagnosticity effect of −2.9% in Study 1a. The results of Study 1 are therefore inconclusive.

## Study 2

In Study 2 we attempted to replicate Experiment 4 in Tversky and Gati (1978) which was reported in Tversky (1977) as the second study providing support for the diagnosticity effect. As in Study 1, the original study consisted of two separate tasks. In the first task participants were asked to categorize sets of countries, while in the second task participants made similarity judgments for these sets of countries.

Because the original study was conducted in the early 70's, the materials were slightly adjusted to accommodate for the fact that some countries no longer exist (see Table 2 for all stimuli). Since the theory behind the diagnosticity effect predicts that the way stimuli are categorized into subsets affects judgments of similarity, the first part of the experiment examines whether the countries are categorized in different subsets, depending on the distractor stimulus that is manipulated between conditions. Only when the inclusion of a distractor country results in a different categorization between conditions in the first categorization task (which can thus be regarded as a manipulation check) is it expected that this specific stimulus affects similarity judgments in the second part. Two replication studies were performed, one with a Dutch sample (Study 2a) and one with an American sample (Study 2b).

**Table 2:** **Overview of categorization data in Study 2a and 2b**.

**Stimuli**						**Sample**			
	**Countries(both versions)**			**Distractor**		**Dutch students**		**mTurk**	
**Trial**	**a—Top**	**b—Left**	**c—Right**	***p* – cond. 1**	***q* – cond. 2**	***b*(*p*) – *b*(*q*) (%)**	***c*(*q*) – *c*(*p*) (%)**	***b*(*p*) – *b*(*q*) (%)**	***c*(*q*) – *c*(*p*) (%)**
1	Russia	Poland	China	India	Hungary	71.05	69.53	68.47	43.97
2	England	Iceland	Belgium	Switzerland	Madagascar	20.00	30.77	27.04	20.77
3	Bulgaria	Czech Republic	Serbia	Greece	Poland	14.13	13.15	17.04	10.91
4	America	Brazil	Japan	China	Argentina	54.83	61.27	39.01	31.98
5	Cyprus	Greece	Crete	Malta	Turkey	13.66	36.18	8.64	10.70
6	Sweden	Finland	Netherlands	Switzerland	Iceland	17.93	19.51	8.15	12.09
7	Israel	England	Iran	Syria	France	46.15	81.56	54.46	83.10
8	Austria	Sweden	Hungary	Poland	Norway	49.39	57.18	25.57	45.23
9	Iran	Turkey	Kuwait	Iraq	Pakistan	6.15	16.92	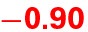	21.82
10	Japan	China	Germany	America	North-Korea	58.69	48.28	55.23	39.83
11	Uganda	Libya	Zaire	Angola	Algeria	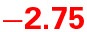	29.95	11.92	19.51
12	England	France	Australia	New-Zeeland	Italy	77.20	77.23	50.49	31.05
13	Venezuela	Colombia	Iran	Kuwait	Brazil	74.08	33.24	80.24	24.91
14	Serbia	Hungary	Greece	Turkey	Poland	43.61	33.19	12.24	8.04
15	Libya	Algeria	Syria	Jordan	Tunis	14.22	26.60	25.75	25.85
16	China	Russia	India	Indonesia	America	45.17	45.31	17.49	8.82
17	France	Germany	Italy	Spain	England	57.20	39.30	45.26	12.89
18	Cuba	Haiti	North-Korea	Albania	Jamaica	24.69	9.67	37.56	11.99
19	Luxembourg	Belgium	Monaco	San Marino	Netherlands	67.95	64.97	52.16	28.82
20	Serbia	Czech Republic	Austria	France	Poland	40.48	19.60	15.85	7.18

*The categorization and similarity results represent the difference (in percentages) of choices for that target b and c between the two conditions (p and q). The right most column represents the average difference (in percentages) between the two conditions in the number of times option b was ranked higher than c. Results in green are in the direction predicted by a diagnosticity effect, those in red are opposite to the expected direction*.

### Study 2a

#### Method

***Categorization task***. For the categorization task, 131 students from Eindhoven University of Technology were approached on the campus and presented with a small booklet in which 20 groups of four countries were displayed. Following Tversky and Gati (1978) in each trial participants were asked to create two different groups of countries. Similarly to Study 1, three of the countries were the same across condition (*a, b*, & *c*) with a fourth distractor country varying across conditions (*p* or *q*).

**Figure 5:**
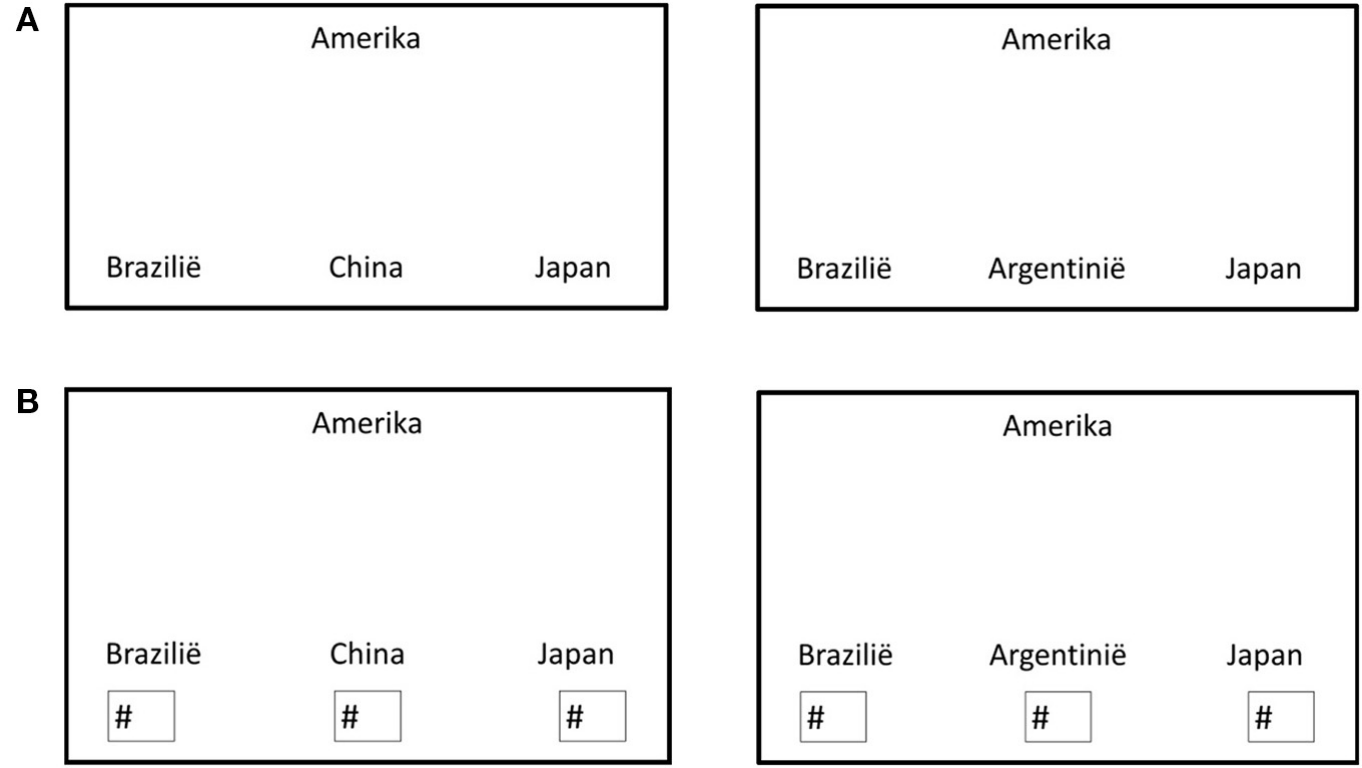
**(A)** Example of a similarity-judgment trial completed by the Dutch participants in condition 1 (left) and condition 2 (right). **(B)** Example of a ranking trial completed by Dutch participants in condition 1 (left) and condition 2 (right).

***Similarity task***. The similarity task was conducted at the social psychology lab in Tilburg University (*N* = 198) and was the first task in a series of unrelated experiments. Participants were presented with a booklet containing both judgment tasks. On the first pages, 20 trials were printed in which the four countries were presented with country *a* on top, and three other countries (*b, p*/*q, c*) at the bottom (see Figure 5A for an example trial). They were asked to indicate which country was most similar to the country printed at the top. Subsequently, the 20 trials were presented a second time, only now participants were asked to rank the countries at the bottom in order of similarity to the country at the top (see Figure 5B).

#### Results

***Categorization task***. It was expected that distractor country *p* would be more likely to be grouped with *c* than distractor country *q*. In other words, in condition 1 it was expected that most participants would categorize country *a* and *b* in one group, and country *p* and *c* in the other group. In condition 2 it was expected that participants would be more likely to categorize country *a* and *c* together, and country *b* and *q* together. In 19 of the 20 trials, participants were more likely to group *a* and *b* together in condition 1 as compared to condition 2. In all 20 trials, participants were more likely to group country *a* and *c* together in condition 2 as compared to condition 1 (see Table 2). Note that one trial (trial 11; Uganda, Libya and Zaire with Angola or Algeria) did not show the desired categorization effect on *b*. Because the diagnosticity effect is theorized to be the result of influences of categorization on similarity judgments, a lack of a categorization effect is the equivalent of a failed manipulation check, and means no diagnosticity effect is expected.

***Similarity task***. Judgments of similarity were expected to follow the categorization task, such that participants in condition 1 would be more likely to indicate country *b* was similar to country *a* as compared to participants in condition 2, while participants in condition 2 would be more likely to indicate country *c* was similar to country *a* as compared to participants in condition 1. For clarity, we will refer to results in predicted direction a “positive” diagnosticity effect. Results showing an opposite pattern from predictions will be referred to as “negative” diagnosticity effects.

For the 19 trials testing for a diagnosticity effect on country *b*, 12 showed a positive diagnosticity effect, 1 trial had an effect of exactly 0, and 6 trials showed a negative diagnosticity effect (opposite to predictions). The average effect across all 19 trials was 5.6%. Effects for country *c* were similar, out of the 20 trials 13 showed a positive effect, 1 an effect of exactly 0, and 6 a negative effect opposite to predictions, the average diagnosticity effect was 9.8%. Overall, the average diagnosticity effect was 7.79% (*SD* = 14.10), *t*_(38)_ = 3.45, *p* = 0.001, *g_av_* = 0.31, 95% CI [0.12, 0.50], JZS BF_10_ = 19.77.

These findings, however, could be the result of a mere substitution effect. Similar to Study 1, rank orders were categorized as either indicating country *b* was more similar to country *a* (at the top) than country *c* was to country *a*, or the other way around^5^. In other words, participants were categorized as either placing country *b* higher in the ranking than country *c*, or ranking country *c* higher than country *b*. Eliminating substitution-effects resulted in no indication of a diagnosticity effect (see Table 2). Out of 19 trials, 12 showed a negative effect of categorization and 7 a positive effect. Overall the effect of categorization was −1.21% (*SD* = 6.04), *t*_(18)_ = −0.87, *p* = 0.40, *g_av_* = −0.05, 95% CI [−0.15, 0.06], JZS BF_10_ = 0.25. This means that participants were slightly less likely (but not significantly so) to rank country *b* above country *c* in in condition 1 as compared to condition 2. For an overview of the similarity judgments, see Table 3.

**Table 3:**
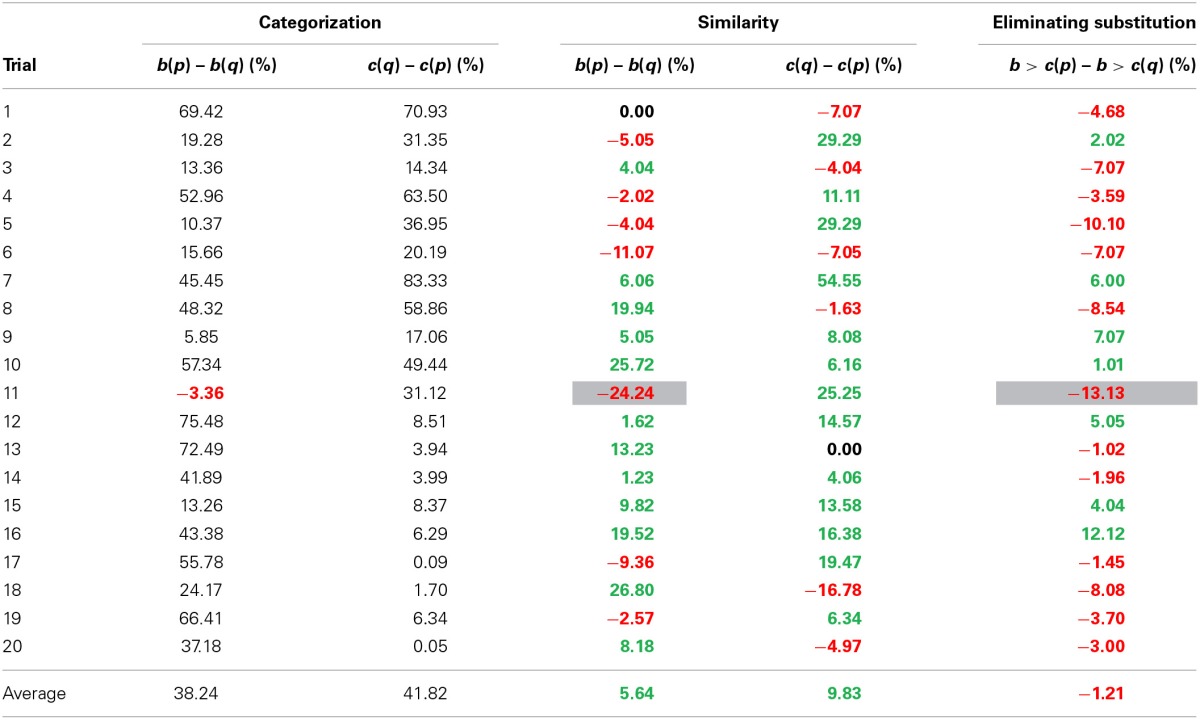
**Results Study 2a**.

*The categorization and similarity results represent the difference in percentage selecting for that target between the two conditions (p and q). The right most column represents the average difference (in percentages) between the two conditions in the number of times option b was ranked higher than c. Results in green are in the direction predicted by a diagnosticity effect, those in red are opposite to the expected direction. Values with gray background are excluded when calculating the average over trials*.

### Study 2b

#### Method

For the categorization task, 153 workers on Amazon mTurk completed 20 trials similar to those in Study 2a, but translated into English.

The similarity task was completed by 201 different workers on mTurk and consisted of 40 trials. The task was identical to Study 2a, except for the English translation.

#### Results

***Categorization task***. Similar to Study 2a, it was expected that most participants would group country *a* and *b* together, as well as group country *p* and *c*. In condition 2 it was expected that participants would be more likely to group country *a* and *c* together, as well as group country *b* and *q*. In condition 1, the prediction was confirmed for 19 of the 20 trials (trial 9, Iran, Turkey, Kuwait and Pakistan or Iraq did not show the expected categorization effect on *b*, which was a different trial than in Study 2a)^6^. For an overview of the categorization data, see Table 2. In condition 2, participants were more likely to group country *a* and *c* together in condition 2 as compared to condition 1 for all 20 trials. Since categorization did not show the desired effect for one trial in condition 1, this trial will be excluded from subsequent analyses.

***Similarity task***. Based on the diagnosticity principle, it was predicted that participants would be more likely to indicate country *b* was similar to the target (country *a*) in condition 1 as compared to condition 2. Furthermore, it was predicted that participants in condition 2 would be more likely to indicate country *c* was most similar to country *a*. Results consistent with these predictions are described as positive diagnosticity effects whereas those in opposite direction are referred to as negative diagnosticity effects.

One trial did not show the required categorization effect on country *b*. This is the equivalent of a failed manipulation check, which means that for that trial no diagnosticity effect is predicted. Therefore, only 19 trials were analyzed. In 12 of the 19 trials a positive diagnosticity effect was found, while in 6 of the 19 trials a negative diagnosticity effect was observed. The average diagnosticity effect across all 19 trials was 1.2%. For country *c*, 13 of the 20 trials showed a positive diagnosticity effect, 6 a negative one. The average effect across all 20 trials was 7.4%. Overall, there was some indication of a diagnosticity effect of 4.4% (*SD* = 13.32), *t*_(38)_ = 2.06, *p* = 0.046, *g_av_* = 0.24, 95% CI [0.00, 0.49], but this difference was not convincing when evaluated with Bayesian statistics, JZS BF_10_ = 0.89.

To examine whether the diagnosticity effect was caused by a substitution effect, we analyzed the rank orders following Medin et al. (1995). Eliminating substitution reduced the differences between conditions. Out of 19 trials, 12 showed positive diagnosticity effect on similarity judgments (indicating people in condition 1 were more likely to rank country b higher than country *c*) and 7 showed a negative diagnosticity effect. Overall the effect of categorization on similarity judgments was 1.24% (*SD* = 6.50), *t*_(18)_ = 0.83, *p* = 0.42, *g_av_* = 0.06, 95% CI [−0.09, 0.22], JZS BF_10_ = 0.24. This means that participants were slightly more likely to rank country *b* above country *c* in condition 1 as compared to condition 2, but this difference was not statistically significant. For an overview of the similarity judgments, see Table 4.

**Table 4:**
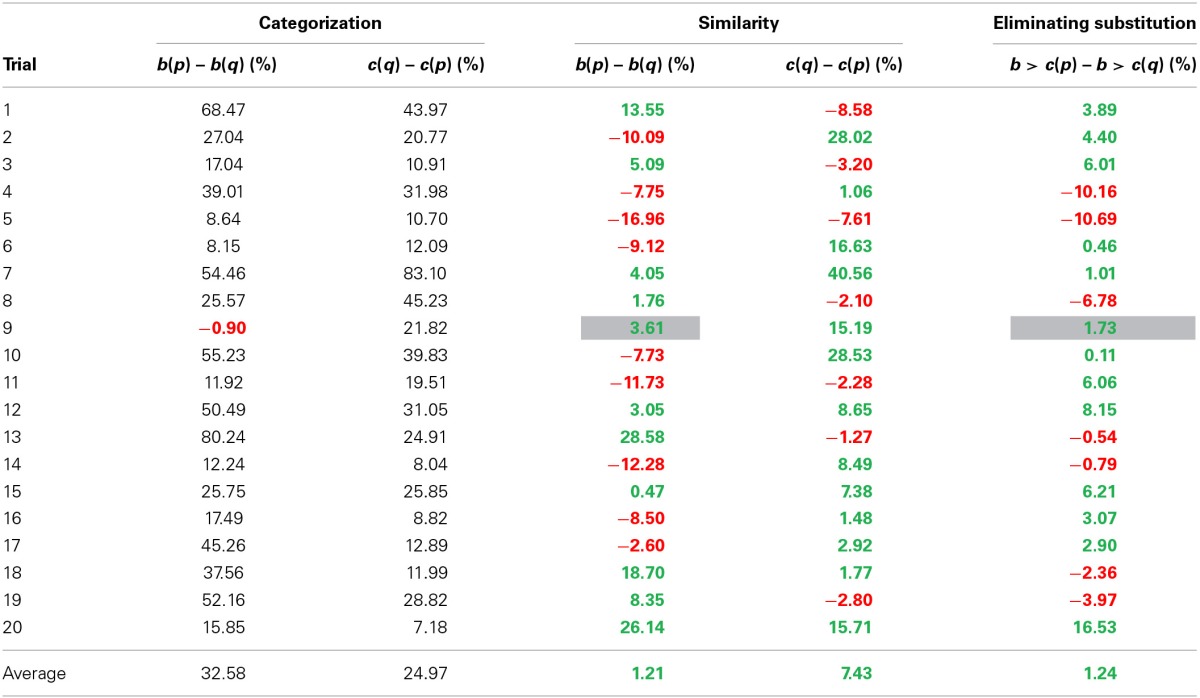
**Results Study 2b**.

*The categorization and similarity results represent the difference (in percentages) in choices for targets b and c between the two conditions (p and q). The right most column represents the average difference (in percentages) between the two conditions in the number of times option b was ranked higher than c. Results in green are in the direction predicted by a diagnosticity effect, those in red are opposite to the expected direction. Values with gray background are excluded when calculating the average over trials*.

## Meta-analysis

To evaluate the overall support for an effect on the similarity task and the ranking task (which controls for substitution effects) we performed two meta-analyses. For Studies 1a and 1b we calculated odds ratios and transformed these to Cohen's *d* following the recommendations by Borenstein et al. (2009). For the two analyses of the similarity task in Study 1a and 1b (i.e., the difference in choices for option *b* between conditions, and the differences in choices for option *c* between conditions) a combined effect size was calculated (with *r* = 1 for the variance calculations because changes in one test are inextricably linked to the outcome in the other test). For the similarity task in Study 2a and 2b Cohen's *d* was calculated based on the difference in choices for *b* and *c* depending on the distractor in each of the two conditions (over the 39 difference scores). For the ranking task in Study 2a and 2b Cohen's *d* was calculated based on the change in ranking (over the 19 difference scores). The analyses were performed in R using the meta package (Schwarzer, 2007). The R script and data for the meta-analysis are available from http://osf.io/e6cr3/.

**Figure 6:**
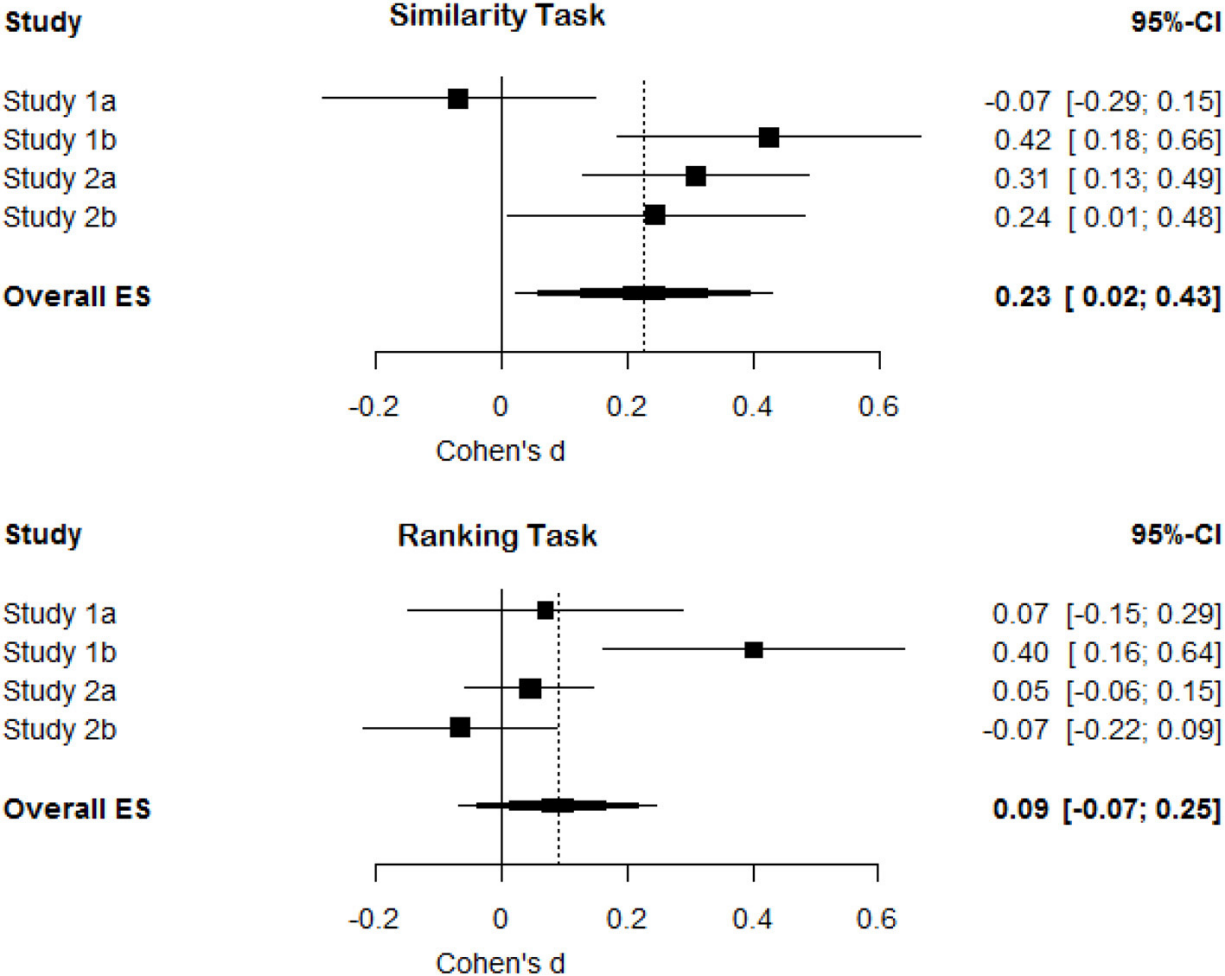
**Forest plots for effect sizes (Cohen's *d*) and their confidence intervals in Study 1a, 1b, 2a, and 2b and the overall meta-analytic effect size for the similarity task and the ranking task (controlling for substitution effects)**.

A random-effects meta-analysis of the similarity task revealed an overall effect size of *d* = 0.23, 95% CI [0.02, 0.43] that differed statistically from zero, *p* = 0.03, and some heterogeneity in the effect sizes *Q*_(3)_ = 10.57, *p* = 0.01, τ^2^ = 0.03%, *I*^2^ = 71.6% (mainly due to the smaller effect size in Study 1a). This means we replicate the pattern of results as observed by Tversky (1977), although the size of the effect is substantially smaller (see Figure 6, top pane). A random-effects meta-analysis of the ranking task, which controls for substitution effects, revealed an overall effect size of *d* = 0.09, 95% CI [−0.07, 0.25]. This effect size did not differ statistically from zero, *p* = 0.26. There was again heterogeneity in the effect sizes *Q*_(3)_ = 10.38, *p* = 0.02, τ^2^ = 0.02%, *I*^2^ = 71.1% (mainly due to the larger effect size in Study 1b). This indicates that after controlling for substitution effects, we did not observe support for a diagnosticity effect (see Figure 6, bottom pane).

## General discussion

In four studies we examined the presence of a diagnosticity effect. It was predicted that judgments of similarity would reflect choices of categorization. This means that in all studies, participants in condition 1 (e.g., with two frowning and one smiling face as choice options) would judge item *b* (e.g., a smiling face) to be more similar to the target (e.g., a neutral face) as compared to participants in condition 2. Furthermore, participants in condition 2 (e.g., with two smiling faces and one frowning face as choice options) should judge item *c* (e.g., a frowning face) to be more similar to a target than participants in condition 1 (see Figure 1).

As in Tversky (1977) and Tversky and Gati (1978) we found significant differences in similarity judgments as a function of the distractor stimulus in Studies 1b, 2a, and 2b (but no effect on similarity judgments in Study 1a). The results in Study 1b are similar, though smaller than those found by Tversky (1977). In Study 1b, we found a diagnosticity effect of 14% and 23% (meaning that in condition 2, compared to condition 1, choice for item *b* was decreased by 14% and choice for item *c* increased by 23%). The effects originally reported by Tversky (1977) were 32% for *b* and 38% for *c*. The results of Study 2a, and 2b are also very similar to those originally observed by Tversky and Gati (1978), but again revealed smaller effects. In the original work, Tversky and Gati (1978) found diagnosticity effects of approximately 11.8% on choices for *b* and 11.6% on choices for *c*. In our studies we found an effect of 5.6% and 1.2% on choices for *b* (resp. study 2a and 2b) and 9.8% and 7.4% on choices for *c*. As such, our experiments result in similar, but less strong, differences in similarity judgments as in Tversky and Gati's original experiments (Tversky, 1977; Tversky and Gati, 1978). Thus, as the meta-analysis indicates, without eliminating possible substitution effects we replicate the original findings by Tversky (1977) and Tversky and Gati (1978).

However, eliminating substitution effects strongly reduces the observed differences between conditions with averages of 2.89% (1a), 17.5% (1b), −1.21% (2a), and 1.24% (2b). Overall the size of differences between conditions, when substitution effects were eliminated, clustered around zero (see Figure 6). The results of Study 1b may seem to be an exception to this overall pattern by revealing a significant positive diagnosticity effect even after substitution effects are eliminated. Although the observed effect in Study 1b is surprising assuming that the null-hypothesis is true, we have no clear explanation for the presence of an effect in this single trial, but the absence of an effect in the other studies. The two most probable interpretations seem to be that the diagnosticity effect does not exist and study 1b is a false positive, or that unknown moderators exist. We will now discuss these possibilities.

One possible interpretation of these results is that Tversky's original findings on the diagnosticity principle are indeed confounded by a substitution effect. The diagnosticity principle is only a part of the contrast model developed by Tversky (1977). The main goal of Tversky's (1977) work on similarity on context was to demonstrate that subjective similarity judgments did not always correspond to predictions made by geometrical models of similarity. He highlighted the role of context and attention in similarity judgments, and the core of Tversky's contrast model, that the similarity between objects is expressed as a function of their common and disjunctive features, remains a powerful idea in cognitive psychology (for a review, see Goldstone and Son, 2005).

If we assume that diagnosticity effects do not exist, the differences found in Study 1b are surprisingly large. It could be the case that categorization indeed influences judgments of similarity, but that it does so in a more complex way suggesting that the model needs to be adapted rather than rejected. As such, our findings are relevant for more recent models of similarity that try to account for the effects observed by Tversky (1977) in geometric models of similarity (e.g., Pothos et al., 2013).

### Potential moderators

It is possible that diagnosticity has an effect on judgments of similarity, but that its influence is more complex than previously believed. Two of these moderators (shared (mis)matches and ranking vs. selecting) cannot explain the null-findings reported in this manuscript. The third moderator (semantic vs. objective features) might. These three moderators are discussed below.

**Figure 7:**
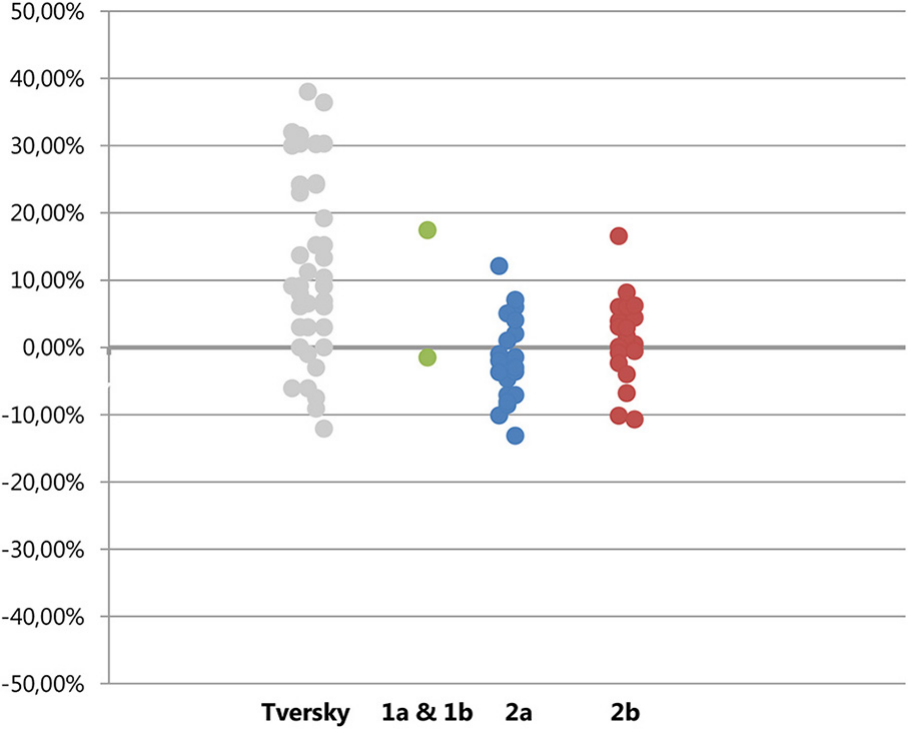
**Plot of diagnosticity effects found by Tversky (left column) and those in Experiment 1, 2a, and 2b**. Absolute differences are plotted for Tversky's data, for Study 1a, 1b, 2a, and 2b the ranking-values (eliminating substitution effects) are plotted.

Goldstone et al. (1997) tried to build on Tversky's model by making a distinction between shared matches and shared mismatches, and predicted that shared matches would lead to opposite effects on judgments of similarity from shared mismatches. If half of the trials in Study 2 contained of shared matches and the other half of shared mismatches, this could possibly explain the average effect of zero. Inspecting the data, this explanation seems unlikely. As can be seen in Figure 7, the sizes of the diagnosticity effect seems to be spread around zero rather than form two clusters, one above zero (for the trials featuring a mismatch) and one below (for the trials featuring a match).

**Figure 8:**
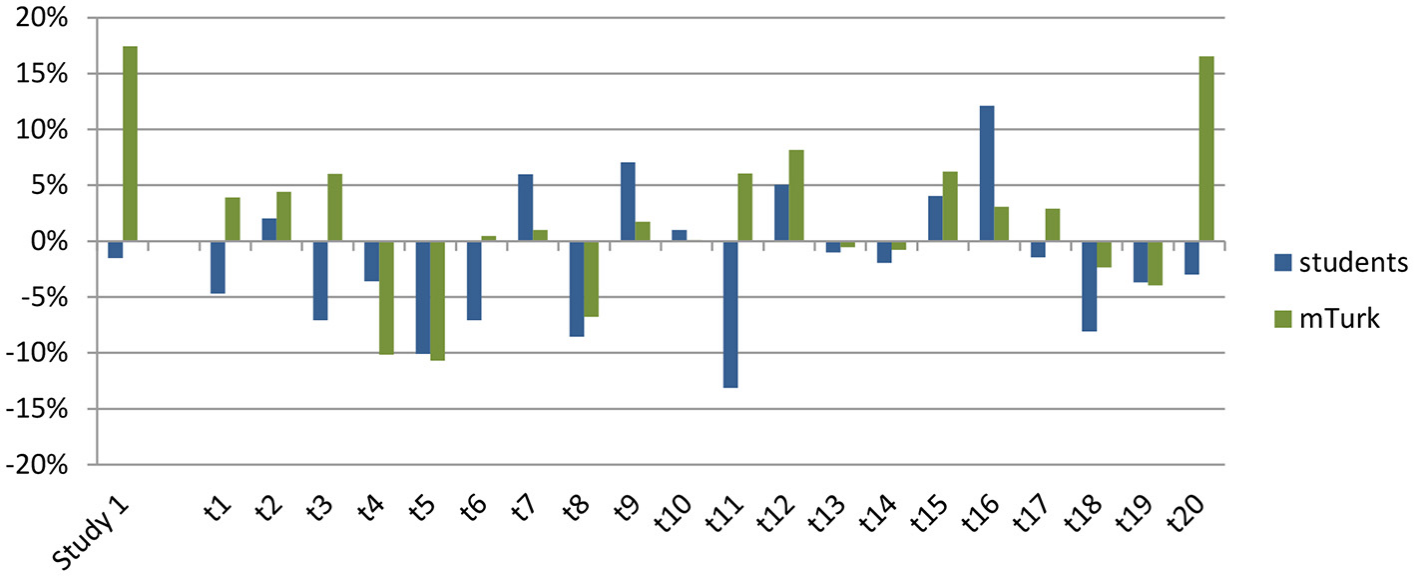
**Size of the diagnosticity effect for each individual trial (on the x-axis) for the students (in blue, studies 1a and 2a) and mTurk (in green, studies 1b and 2b) samples**.

Further evidence against this idea comes from the categorization data. In the Dutch sample, all participants created only groups of two countries. If the distractor country was perceived as sharing a matching feature with the target and one of the choice-options, participants would have created groups of three and one instead of two groups of two. In the mTurk sample, participants did on occasion create groups of three countries. However, this grouping did not relate to the size and direction of the diagnosticity effect, the correlation between amount of participants making clusters of three and the diagnosticity-effect was smaller than 0.01, see supplementary materials on http://osf.io/e6cr3/. Finally, if the distinction between shared matches and shared mismatches would explain the average effect of zero, this implies that the effects should be consistent between Study 2a and 2b. As can be seen in Figure 8, this does not seem to be the case.

It is also possible that forcing participants to rank the options caused them to approach the similarity task in a different way. Even though we cannot fully reject this possibility, it seems unlikely that this is the case. Because participants first selected one of the three stimuli, and subsequently rank-ordered all three stimuli, we can test for consistency in answers between their choice for the most similar stimulus in the first part of the task, and the stimulus rank-ordered as the most similar stimulus. As can be seen in the supplementary materials, there were very few instances in which participants did not rank their previous choice of being most similar as number 1. One could of course argue that by first completing the choice trials and then completing the ranking trials, participants tried to be extra consistent. However, if participants tried to be consistent this would increase the likelihood of finding a diagnosticity effect rather than decrease it. Furthermore, Tversky(1977, p. 343) reports that changing the similarity-judgments by asking participants to rank order the options did not change his results. Note that those data were analyzed in the same way as the choice-studies (only analyzing the item ranked as most similar rather than analyzing whether *b* was ranked higher or lower than *c*) and did not eliminate substitution as a confound.

The trials used in Experiment 1, and in conceptual replications by Medin et al. (1995) and Goldstone et al. (1997) were all trials in which participants judged the stimuli on objectively observable features such as the shape of a mouth or the size of a triangle. The trials in Study 2 on the other hand used countries for which participants were expected to create categories on the basis of their subjective knowledge about the features of the countries. It is possible that the automatic categorization that is supposed to influence judgments of similarity only happens when participants can directly see and compare these features. Because the results presented in Tversky (1977) are confounded with substitution effects, it is possible that he interpreted only a substitution effect as evidence for diagnosticity in Study 2, using countries, but that the results of Study 1, using faces, actually do (partially) reflect a diagnosticity effect. This would also be consistent with the findings by Tversky (1977) who, using the faces-task, found such large differences in choices that even when a very conservative test that overcorrects for substitution effects is used, his data still show significant differences in choice proportions (see page 6 in Medin et al., 1995 for a more extensive discussion) Since the original study using the faces can readily be replicated, it would be useful if more people would administer this experiment to their participants (materials can be downloaded from http://osf.io/e6cr3/) to see whether the effects replicate. The results could be shared among researchers through for example psych file-drawer and will allow the field as a whole to gain more insights in whether and when diagnosticity effects emerge.

## Conclusion

To conclude, in 42 trials spread over 4 studies we found differences in choice proportion similar to those found by Tversky (1977). After eliminating substitution effects as a possible confound, these effects largely disappeared. There are two explanations that seem likely to explain this difference. One possibility is that there really are no diagnosticity effects and previously found differences have been substitution-effects wrongfully interpreted as diagnosticity effects. The other explanation could be that diagnosticity effects do exist but only in situations where the features of the targets are visual and readily available rather than dependent on subjective and semantic knowledge.

### Conflict of interest statement

The authors declare that the research was conducted in the absence of any commercial or financial relationships that could be construed as a potential conflict of interest.

## Footnotes

^1^The first category therefore consisted of the 3 possible rankings {b>p>c, p>b>c, and b>c>p}, the second category consisted of the 3 possible rankings {c>p>b, p>c>b, and c>b>p}.

^2^One participant did not fully complete the ranking measure.

^3^Due to an error, only 200 participants were collected in Study 1b, rather than the planned 250. This means power was slightly lower than intended.

^4^Three participants did not correctly complete the ranking part.

^5^The first category therefore consisted of the 3 possible rankings {b>p>c, p>b>c, and b>c>p}, the second category consisted of the 3 possible rankings {c>p>b, p>c>b, and c>b>p}.

^6^It is possible that trial 11 in study 2a and trial 9 in study 2b did not show the intended categorization effect due to cultural differences. Alternatively since a total of 40 categories had to be created in each study, this could also be a false negative.
